# Two new species of *Cylindrolobus* (Orchidaceae) from the eastern Himalayas

**DOI:** 10.3897/phytokeys.130.33989

**Published:** 2019-08-29

**Authors:** Ji-Dong Ya, Xiao-Hua Jin, Cheng Liu

**Affiliations:** 1 Germplasm Bank of Wild Species, Kunming Institute of Botany, Chinese Academy of Sciences, Lanhei Road 132, Heilongtan, Kunming, Yunnan, 650201, China Kunming Institute of Botany, Chinese Academy of Sciences Kunming China; 2 State Key Laboratory of Systematic and Evolutionary Botany and Herbarium, Institute of Botany, Chinese Academy of Sciences, Nanxincun20, Xiangshan, Beijing, 100093, China Institute of Botany, Chinese Academy of Sciences Beijing China

**Keywords:** Taxonomy, Orchidaceae, *
Cylindrolobus
*, new species, China

## Abstract

Two new species, *Cylindrolobus motuoensis* and *C. glabriflorus* (Orchidaceae), from Southwestern China and north of Myanmar are described and illustrated with detailed photos. *Cylindrolobus motuoensis* is morphologically similar to *C. gloensis* and *C. foetidus*, but can be distinguished from them by having amplexicaul sterile bracts, dark red floral bracts, white flowers, falcate-lanceolate lateral sepals and central keel of lip running from base to the tip of mid-lobe. *Cylindrolobus glabriflorus* is similar to *C. hegdei* and *C. tenuicaulis* but differs from them by having longer and wider leaves, obovate bracts, and the reddish brown central papillate keel of lip.

## Introduction

The genus *Cylindrolobus* Blume consists of 60‒70 species, distributed in the tropical region from East Himalaya, China, Southeast Asia to New Guinea (Chen et al. 2009, Ormerod 2014, Ng et al. 2018). *Cylindrolobus* was originally proposed as a section of *Eria* Lindl., Pridgeon et al. (2005) suggested that *Cylindrolobus* should be subsumed into *Callostylis* Blume. Recent molecular and morphological studies suggest that *Cylindrolobus* is a distinct genus, characterized by a multi-noded stem with leaves at apex, short inflorescences with one to several flowers and conspicuous and colorful bracts (Chen et al. 2009, Ng et al. 2018).

## Materials and methods

Living plants were collected from Xizang (Tibet) Autonomous Region of China and north of Myanmar during the botanical expeditions in 2016 and in 2018. The shapes, colors of flowers and other details of the plants observed, measured and photographed, as well as specimens collected, were based on living materials from 2017 to 2019. Morphological photographs of the lip, column and pollinia were taken using an Olympus SZX16. All voucher specimens were deposited in KUN (Herbarium of Kunming Institute of Botany, Chinese Academy of Sciences).

## Taxonomic treatment

### Cylindrolobus motuoensis

Taxon classificationPlantaeAsparagalesOrchidaceae

X.H.Jin & J.D.Ya sp. nov.

7FEB02BF41FA53FAAA25C77385AD843B

urn:lsid:ipni.org:names:60479344-2

Figures 1 , 3A

#### Diagnosis.

*Cylindrolobus motuoensis* is similar to *C. gloensis* (Ormerod & Agrawala) Schuit., Y.P. Ng & H.A. Pedersen, and *C. foetidus* (Aver.) Schuit., Y.P. Ng & H.A. Pedersen in terms of morphological structure and shape of the flowers (Hu et al. 2010, Agrawala and Ormerod 2014, Ng et al. 2018). The new species can be distinguished from *C. gloensis* by the smaller flowers, elliptic and concave bracts, and ovate lip with three keels, mid-lobe thickened and papillate on margin. The new species can be distinguished from *C. foetidus* with longer and wider leaves, dark red and elliptic floral bracts, white flowers and falcate-lanceolate lateral sepals.

#### Type.

CHINA. Xizang Autonomous Region: Motuo, subtropical evergreen broad-leaved forest, alt. 2000 m, 26 Feb 2017, *Ji-Dong Ya, Cheng Liu, Hua-Jie He 17HT0073* (holotype: KUN!).

#### Additional specimen examined.

CHINA. Xizang Autonomous Region: Motuo, subtropical, evergreen broad-leaved forest, 26 Feb 2017, *Xiao-Hua Jin, Ji-Dong Ya, 17HT1088* (paratype: KUN!)

#### Description.

Epiphytic herb. Roots terete, slender, pubescent, *ca.*1.0‒1.5mm thick. Rhizome creeping, to 3‒4 mm thick. Stem terete, slender, 3(2) leaved apically, covered by close-fitting sheaths, 18‒24 cm long, 3‒6 mm thick. Leaves ligulate-lanceolate, acuminate, 10‒13 cm long, 1.5‒2.0 cm wide. Inflorescences axillary, pubescent, borne on near the apical of the stem, 2‒3 cm long, 2 flowered; peduncle 1.0‒1.5 cm long; 2 sterile bracts, smaller, amplexicaul; rachis 0.2 cm long, floral bracts dark red, elliptic, acute, concave, sparsely tomentum, 7 mm long, 3 mm wide. Flowers white, sepal externally with brown tomentum, peduncle and ovary ca. 1.0‒1.5 cm long, densely brown tomentum. Dorsal sepal lanceolate, acute, 5 veined, 11 mm long, 4 mm wide; lateral sepals falcate-lanceolate, acute, 5 veined, 9 mm long, 5 mm wide, base adnate to column foot form a subglobose and obtuse mentum; petals lanceolate, slightly oblique, acute, 3 veined, 10 mm long, 3 mm wide; labellum ovate in outline, 3-lobed, base hinged to the apex of the column foot, apex obtuse and emarginate, curved, *c.* 6 mm long, 3 mm wide; lateral lobes suberect, subovate, apex slightly introvert; mid-lobe ligulate, ca. 3 × 3 mm, thickened and papillate on margin, apex emarginate; disk with 3 keels, central keel longitudinal thickened, with orange papilla, running from base to the tip of mid-lobe, lateral keels glabrous, running from base to middle of mid-lobe. Column semiterete, ca. 4 mm long, broad winged at ventrally; foot incurved, ca. 3.5 mm. Anther cap ovate, ca. 1 mm × 1 mm, pollinia 8, yellowish white, compressed rectangular, anterior ca. 0.5 mm × 0.4 mm × 0.2 mm, posterior 4 smaller. Fl. February-March.

**Figure 1.**
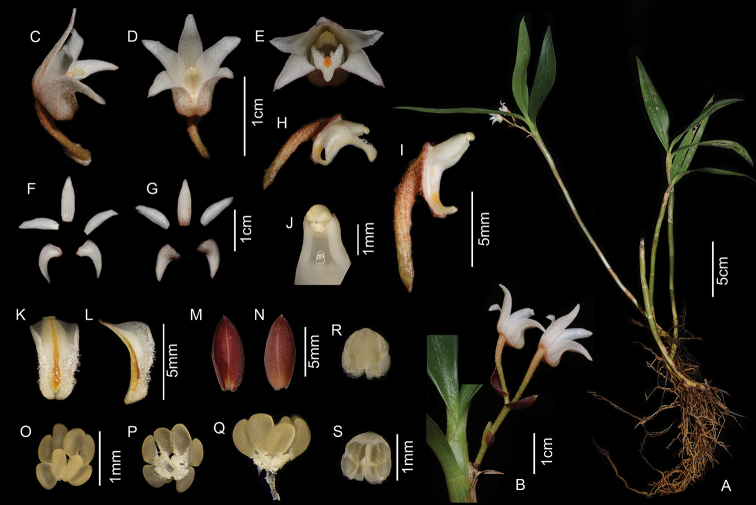
*Cylindrolobus motuoensis* X.H.Jin & J.D.Ya. **A** Plant **B** Inflorescence **C** lateral view of flower **D** ventral view of flower **E** front view of flower **F** adaxial sepals and petals **G** abaxial sepals and petals **H** lateral view of column and lip **I** lateral view of column **J** front view of column **K** front view of labellum **L** lateral view of labellum (rip cutting) **M** adaxial bract **N** abaxial bract **O** polar view of pollinarium **P** ventral view of pollinarium **Q** lateral view of pollinarium **R** adaxial anther cap **S** abaxial anther cap (Photographed by J.-D. Ya).

#### Etymology.

The new species is named after Motuo, Xizang Autonomous Region of China, where it was discovered in a subtropical evergreen broad-leaved forest.

#### Vernacular name.

Mo Tuo Zhu Lan (墨脱柱兰).

### Cylindrolobus glabriflorus

Taxon classificationPlantaeAsparagalesOrchidaceae

X.H.Jin & J.D.Ya sp. nov.

66A0F1EF8C185284ADFBAD53792D4500

urn:lsid:ipni.org:names:60479345-2

Figures 2 , 3B

#### Diagnosis.

*Cylindrolobus glabriflorus* is similar to *C. hegdei* (Agrawala & H. J. Chowdhery) A. N. Rao and *C. tenuicaulis* (S. C. Chen & Z. H. Tsi) S. C. Chen & J. J. Wood (Agrawala and Chowdhery 2008, Chen et al. 2009). The new species can be distinguished from *C. hegdei* by the longer and wider leaves, glabrous inflorescence, smaller and yellow flowers, yellowish green and obovate bracts and reddish brown central papillate keel of lip. It differs from *C. tenuicaulis* by having longer and wider leaves, longer inflorescence, yellowish green and obovate bracts, longer lip with three calli, lateral margins of mid-lobe thickened and erect.

**Figure 2.**
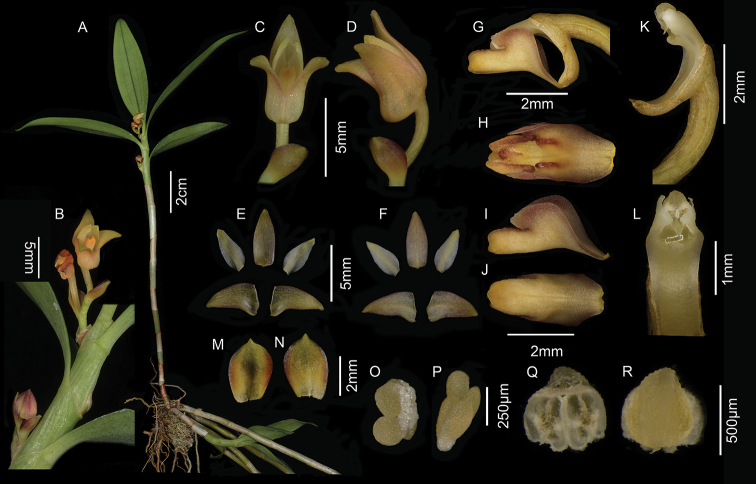
*Cylindrolobus glabriflorus* X.H.Jin & J.D.Ya. **A** Plant **B** inflorescence **C** ventral view of flower **D** lateral view of flower **E** adaxial sepals and petals **F** abaxial sepals and petals **G** lateral view of column and lip **H** front view of labellum **I** lateral view of labellum **J** ventral view of labellum **K** lateral view of column **L** front view of column **M** adaxial bract **N** abaxial bract **O** lateral view of pollinarium **P** polar view of pollinarium **Q** abaxial anther cap **R** adaxial anther cap (Photographed by J.D. Ya).

#### Type.

MYANMAR. Kachin State: Putao Township, Hponkanrazi Wildlife Sanctuary, subtropical, evergreen, broad-leaved, humid montane forest, alt. 2200 m, 12 Apr 2018, *Xiao-Hua Jin, Ji-Dong Ya 18HT1618* (holotype: KUN!).

#### Additional specimen examined.

CHINA. Xizang Autonomous Region: Motuo, subtropical, evergreen broad-leaved forest, alt. 1796 m, 2 Apr 2019, *Ji-Dong Ya, Cheng Liu, 18HT2586* (paratype: KUN!). CHINA. Xizang Autonomous Region: Motuo, subtropical, evergreen broad-leaved forest, alt. 1750 m, 7 Apr 2018, *Hong Jiang, Wei-ping Zhang, Zhou-dong Han 07336* (paratype: YAF!).

#### Description.

Epiphytic herb. Roots terete, slender, pubescent, *ca.*0.8‒1.0 mm thick. Rhizome inconspicuous. Stem clustered, terete, slender, 4-leaved apically, covered by close-fitting sheaths, 13‒25 cm long, 3‒4 mm thick. Leaves lanceolate, acuminate, 4.5‒6.5 cm long, 0.8‒1.5 cm wide. Inflorescences axillary, glabrous, arising from the apical of the stem, 1.3 cm long, 2-flowered; peduncle 0.5 cm long; sterile bracts 1‒2, smaller. Flowers yellow, sepal externally reddish yellow, 7‒8mm long; floral bracts yellowish green with red brown edges, obovate, mucronate, concave, glabrous, 4 mm long, 2.5 mm wide; peduncle and ovary ca. 4‒6 mm long. Dorsal sepal lanceolate, obtuse, 3 veined, 6 mm long, 2 mm wide; lateral sepals falcate-ovate, obtuse, 3-veined, 5 mm long, 3.5 mm wide, base adnate to column foot form a subglobose and obtuse mentum; petals oblong-ovate, slightly oblique at base, obtuse, 1 veined, 5 mm long, 2 mm wide; labellum oblong in outline, 3-lobed, base hinged to the apex of the column foot, apex emarginate, ca.3.5 mm long, 1.5 mm wide; lateral lobes suberect, subovate, apex slightly introvert, disk with 2 reddish brown calli; mid-lobe sub-square, ca. 3.3 mm × 2 mm, lateral margins thickened and erect, with a central papillate keel, reddish brown, ca. 0.5 mm high; apex emarginate. Column semiterete, ca. 3.5 mm long, narrow winged at ventrally; rostellum triangle, 0.2 mm, a hook-like protrusion under the stigma; foot incurved, ca. 4 mm; cap subrotund, ca. 0.6 mm × 0.6 mm, papillate and protrude in front; pollinia 8, yellowish white, compressed subrotund from the lateral view, anterior 4 ca. 0.2 mm × 0.2mm × 0.1mm, posterior 4 smaller. Fl. April-May.

#### Etymology.

The specific epithet “*glabriflorus*” refers to glabrous flowers of this new species.

#### Vernacular name.

Zhong Mian Zhu Lan (中缅柱兰).

**Figure 3.**
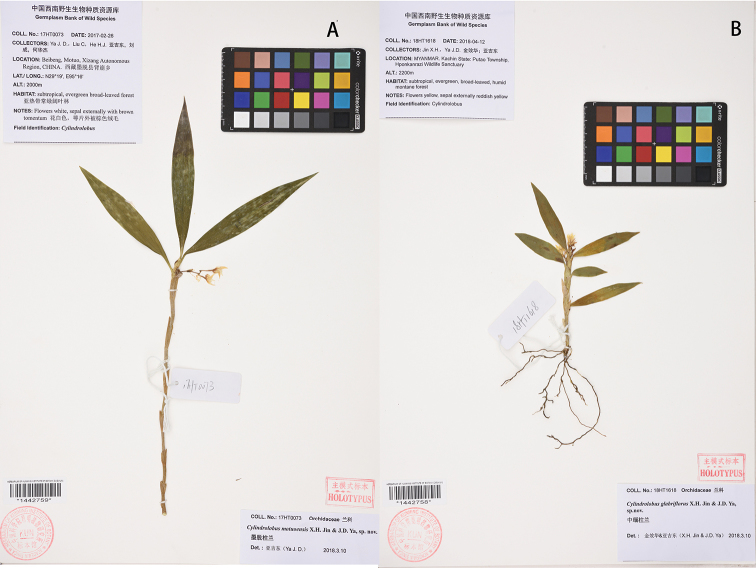
Holotype. **A***Cylindrolobus motuoensis* X.H.Jin & J.D.Ya. **B***Cylindrolobus glabriflorus* X.H.Jin & J.D.Ya.

## Discussion

The generic delimitation of Podochileae Pfitzer has long been confused (Pridgeon et al. 2005, Ng et al. 2018). Recent molecular and morphological evidence reveals that *Erias.l.* should be split into 21 genera, including *Cylindrolobus* which is morphologically characterized by fleshy and elongate stems, lateral inflorescences, conspicuous and colorful bracts.

These two new species are distributed in a narrow area in the border region between China and Myanmar. We found there are many populations of each species and abundant individuals per population during our botanical expeditions. As the habitats of these two species are in a remote location and the border area has restricted access, the effect of human interference and climate change on them is little known. For the time being, these two species are considered as Least Concern (LC) according to current information on these species and the IUCN Red List category (IUCN 2012).

Located at the margin of Qinghai-Tibet Plateau, Motuo is famous for its vertical vegetation system from tropical forest to permanent glacier with elevation approximately 7000 m, which allow the thriving and diversification of plant diversity. In addition, there are many biodiversity hotspots in the eastern Himalayas, e.g. the north of Myanmar, however the species diversity of this region is poorly known. Hence, the species diversity of these border regions requires solid investigations (Liu et al., 2019) and timely conservation action plans in this region, in order to mitigate increasing anthropogenic disturbance and destruction.

The two new species described here increased the members of *Cylindrolobus* in China to seven species (Chen et al. 2009, Hu et al. 2010, Liu et al. 2013, Ng et al. 2018), the key to Chinese species of *Cylindrolobus* are developed here.

### Key to Chinese species of *Cylindrolobus*

**Table:** 

1	Stems stout, clavate	**2**
–	Stems slender, terete	**4**
2	Lip yellow	***C. cristatus***
–	Lip lateral lobes with purple edges, mid-lobe with lighter purple edges and patch	**3**
3	Inflorescence glabrous, the lateral lobes bigger than mid-lobe	***C. clavicaulis***
–	Inflorescence pubescent, the lateral lobes smaller than mid-lobe	***C. marginatus***
4	Inflorescence glabrous	**5**
–	Inflorescence pubescent	**6**
5	lip mid-lobe thickened and papillate on margin	***C. tenuicaulis***
–	lip mid-lobe not thickened and smoothly on margin	***C. glabriflorus***
6	Flowers yellow	***C. foetidus***
–	Flowers white	***C. motuoensis***


## Supplementary Material

XML Treatment for Cylindrolobus motuoensis

XML Treatment for Cylindrolobus glabriflorus

## References

[B1] Agrawala DK Chowdhery HJ (2008) A new species of *Eria* Lindl. (Orchidaceae) from Arunachal Predesh, India. Phytotaxonomy 8: 8–12.

[B2] Agrawala DK Ormerod P (2014) A new species of *Eria* (Orchidaceae) from India under the section Cylindrolobus Taiwania 59(3): 206–209. 10.6165/tai.2014.59.206

[B3] Chen SC Lou YB Vermeulen JJ (2009) *Cylindrolobus* Blume. In: Wu ZY Raven PR Hong DY (Eds) Flora of China (Vol.25). Science Press, Beijing & Missouri Botanical Garden Press, St. Louis, 249–250.

[B4] Hu H Jin XH Sun Q Sun XG (2010) A new record of Orchidaceae from China. Redai Yaredai Zhiwu Xuebao 18: 401–402.

[B5] IUCN (2012) IUCN Red List Categories and Criteria, Version 3.1. (2^nd^ edn). IUCN, Gland.

[B6] Liu JJ Baeten L Verheyen K (2019) Biodiversity on International Borders Requires Solid Inventories. Bioscience 69(5): 322–323. 10.1093/biosci/biz029

[B7] Liu Q Li JW Yin JT (2013) *Eria clavicaulis*, a new record of Orchidaceae from Yunnan, China. Guihuia 33(1): 70–71.

[B8] Ng YP Schuiteman A Pedersen HÆ Petersen G Watthana S Seberg O Pridgeon AM Cribb PJ Chase MW NG YP (2018) Phylogenetics and systematics of Eria and related genera (Orchidaceae: Podochileae). Botanical Journal of the Linnean Society 186(2): 179–201. 10.1093/botlinnean/box088

[B9] Ormerod P (2014) A Synopsis of Eria Lindl. Section Cylindrolobus (Blume) Lindl. (Orchidaceae: Eriinae) in Malesia. Harvard Papers in Botany 19(1): 77–95. 10.3100/hpib.v19iss1.2014.n6

[B10] Pridgeon AM Cribb PJ Chase MW Rasmussen FN (2005) Tribe Podochileae. In: Genera Orchidacearum 4, Epidendroideae (part one). Oxford University Press, Oxford, 529‒596.

